# Retraction Note: MicroRNA-1468 promotes tumor progression by activating PPAR-γ-mediated AKT signaling in human hepatocellular carcinoma

**DOI:** 10.1186/s13046-022-02487-y

**Published:** 2022-09-13

**Authors:** Zhikui Liu, Yufeng Wang, Changwei Dou, Liankang Sun, Qing Li, Liang Wang, Qiuran Xu, Wei Yang, Qingguang Liu, Kangsheng Tu

**Affiliations:** 1 Department of Hepatobiliary Surgery, the First Affiliated Hospital of Xi’an Jiaotong University, 277 Yanta West Road, Xi’an, 710061 China; 2 Department of Hepatobiliary Surgery, Zhejiang Provincial People’s Hospital (People’s Hospital of Hangzhou Medical College), Zhejiang, 310014 Hangzhou China; 3 Key Laboratory of Tumor Molecular Diagnosis and Individualized Medicine of Zhejiang Province, Zhejiang Provincial People’s Hospital (People’s Hospital of Hangzhou Medical College), Zhejiang, 310014 Hangzhou China

**Retraction Note: J Exp Clin Cancer Res 37, 49 (2018)**

**https://doi.org/10.1186/s13046-018-0717-3**

The Editor-in-Chief has retracted this article at the corresponding author's request. After publication, the authors became aware that data from other projects were included in the figures, resulting in image overlap between this article and previous publications from other authors. Specifically:

- Fig. 3a appears to overlap with Fig. 4a in [1];
- Fig. 7g Cyclin D1 lanes 3 and 4 appear highly similar to Fig. 9d E-cadherin lanes 2 and 3 in [2].

Additionally, parts of images in Figs. 2d, 3a, 7g and 8f have subsequently been used in [3–7].

The authors have therefore lost confidence in the data presented in this article.

Author Kangsheng Tu has stated on behalf of all co-authors that they agree to this retraction.
